# Parent–adolescent discrepancies in positive parenting and adolescent problem behaviors in Chinese families

**DOI:** 10.1016/j.heliyon.2024.e25016

**Published:** 2024-01-22

**Authors:** Liuqing Tian, Cong Xin, Yuanxia Zheng, Guoxiong Liu

**Affiliations:** a School of Psychology, Nanjing Normal University, Nanjing, 210097, China; b Institute of Moral Education, Nanjing Normal University, Nanjing, 210097, China

**Keywords:** Positive parenting style, Perceptional discrepancies, Secondary vocational school students, Internalizing and externalizing behaviors, Self-control

## Abstract

Based on the discrepancy–maladaptive hypothesis and general strain theory, in this study, we examined two key aspects: first, the mediating role of self-control in the relationship between parent–adolescent discrepancies in positive parenting and adolescent internalizing and externalizing problems, and second, variations in problem behavior among subgroups with different parent–adolescent dyads reporting patterns. The participants were 349 intact Chinese families, with parents as the primary caregivers and teenagers aged 15–18 years who are attending secondary vocational schools. The results revealed that adolescents generally perceived lower levels of caring and behavioral control than parents. Compared to behavioral control, discrepancies in perceived levels of caring had more significant predicting levels of internalizing and externalizing problems, and the relationship between discrepancies of caring and internalizing and externalizing problems was mediated by self-control. Latent profile analysis revealed three parent–adolescent responding patterns (subgroups); compared to the other subgroups, only the subgroup characterized by adolescents perceiving lower caring and behavioral control than parents exhibited higher levels of internalizing and externalizing problems. The findings of this study provide insights on how parent–adolescent discrepancies may lead to adolescent problem behaviors and highlight the importance of self-control as a mediating mechanism.

## 1 Introduction

Problem behaviors, including internalizing and externalizing problems, encompass a range of atypical behaviors that deviate from social norms, undermine individuals' physical and mental well-being, and impede their social adaptation [1,2]. These behaviors serve as significant predictors of adverse outcomes in adolescent's lives, such as academic underachievement, peer rejection, substance use, and social withdrawal [[2], [3], [4], [5]]. Given the detrimental impact of problem behaviors,^1^ understanding its causes and developing effective prevention strategies are essential [6].

According to the ecological systems theory, the family exerts a far-reaching influence on problem behaviors [7]. Several studies have emphasized the role of positive parenting styles in mitigating adolescents' problem behaviors [8,9]. Parental warmth – a similar structure with caring – can reduce anxiety and depression [[9], [10], [11]]. Higher levels of behavioral control have been reported to decrease internalizing problems and criminal behaviors [10,12,13]. These studies, however, have predominantly considered only the parents' or adolescents' reports of parenting style; no studies have simultaneously considered both perspectives. Evidence shows that discrepancies exist in adolescents' and their parents' reports on parenting^2^ [14]. Such informant discrepancies may yield critical insights into adolescents' development [[15], [16], [17]]. Notably, informant discrepancies have been found to be correlated to worsened adolescent developmental outcomes, such as internalizing (e.g., depression and anxiety) [18,19] and externalizing behaviors (e.g., delinquency) [20,21]. Regrettably, this aspect remains unexplored among Chinese scholars, and parent–adolescent discrepancies in parenting style are simply considered measurement errors [22].

In Chinese culture, parents are always strict but passionate about their offspring, influenced by a traditional ideology featuring an emphasis on collectivism and filial piety [23]. Therefore, they tend to behave with higher levels of physical punishment, harsh discipline, and lower autonomy [24,25]. The past three decades saw China's dramatic economic development and modernization, changing the parents' upbringing beliefs and practices [26]. Zhang et al. [23] found four kinds of parenting styles in Chinese families. The authoritative and authoritarian were similar to Baumrind's classification. The former decreased adolescents' problem behaviors, while the latter increased. About 20 % of parents showed high-level undifferentiated parenting styles, characterizing authoritative parenting with high levels of harshness. Adolescents in this kind of parenting had lower problem behaviors. The rest 40 % of parents showed average-level undifferentiated parenting, featuring no salient characteristic, which undermined adolescents' adjustment and led to more internalizing and externalizing problems. These revealed the influence Chinese culture exerted on parenting and adolescent development. However, because Chinese parents were less likely to express love verbally or discuss affection openly with their adolescents, they may have less effective communication with their offspring, finally enlarging parent–adolescent disagreements and further resulting in adolescents' maladaptive behaviors [18,23,[27], [28], [29]]. Unfortunately, parent–adolescent discrepancies in parenting styles have not been appreciated in China. It is crucial to investigate informant discrepancies and their association with problem behaviors in the context of the Chinese culture.

Therefore, this study aimed to explore the relationship between informant discrepancies in positive parenting styles and adolescents’ problem behaviors in Chinese families. The findings of this study can enable a comprehensive understanding of this phenomenon across different cultural backgrounds.

### 1.1 Characteristics of parent–adolescent discrepancies in positive parenting

Studies on parent–adolescent discrepancies in parenting are limited in China; thus, it is pertinent to first explore their characteristics. Parent–adolescent discrepancies refer to the divergence between parents' and adolescents' reports on parenting; these discrepancies cannot be directly measured but can only be obtained by analyzing the parents' and adolescents’ respective reports [30]. Informant discrepancies can be understood from two perspectives, namely quantitative and qualitative. From a quantitative standpoint, an observed difference score can be derived by subtracting the standardized adolescent scores from the standardized parent scores (Standardized Difference Score) [21,30,31]. This score enhances the accuracy and interpretability of the informant discrepancies; however, it does not provide information about the reliability of specific discrepancy patterns across different samples [32]. From a qualitative standpoint, latent profile analysis (LPA), a statistical method that classifies parent–adolescent dyads (namely, adolescents and their male or female caregiver) into distinct patterns based on their responses to the scales, can capture different types of discrepancies (e.g., adolescent-report > parent-report or vice versa) and address the limitations of the quantitative approach [32,33]. Considering both aspects simultaneously is not only beneficial for generalizing the results, but it also paves the way for applied research [32]. However, only a few researchers have examined both aspects in the same study.

Quantitatively, numerous studies have shown that parents tend to perceive higher levels of positive parenting styles than their adolescent children. Specifically, parents report higher parental warmth, nurturance, and demandingness than their offspring [21,22,31]. In addition, in several indicators of behavioral control, like parental knowledge, monitoring, and discipline, parents still report higher than adolescents [31,34,35]. According to the generational stake hypothesis, parents, driven by intergenerational genetic inheritance, place greater emphasis on raising their descendants, leading them to be more attuned to positive parenting behaviors than their offspring [36]. Additionally, the attribution bias context model suggests that parents, as nurturing providers, tend to attribute positive parenting behaviors to parental factors, perceiving them as stable occurrences caused by their effort. However, adolescents, as parenting recipients, tend to attribute positive parenting behaviors to external factors, resulting in lower perception of positive parenting [37].

Qualitatively, fewer studies have focused on the patterns and characteristics of informant discrepancies. Leung [38] identified four distinct subgroups within the participating families: the convergent-high subgroup, convergent-low subgroup, divergent-medium-low-parent/medium-high-adolescent subgroup, and divergent-medium-high-parent/medium-low-adolescent subgroup. Some other researchers have identified three patterns, namely the no consistent disagreements profile, child consistently over parent profile, and parent consistently over child profile, in some American minority households [20,27]. These studies have shed light on the various patterns of informant discrepancies observed across families.

While researchers have explored informant discrepancies across different nationalities and countries [21,39], no such study has been conducted in mainland China yet. Investigating this phenomenon in the Chinese context is essential because of the unique cultural backdrop shaped by Confucianism, Buddhism, and Taoism, which foster a collectivistic orientation distinct from Western cultures [22]. These cultural disparities give rise to variations in family dynamics and processes within the Chinese context. For instance, although the rapid development of Chinese society in recent decades has changed parenting practices and beliefs [23], training and monitoring are still critical features of parental control in the Chinese culture [40]. Few studies have focused on different patterns in informant discrepancies, thus hindering a comprehensive understanding of informant discrepancies. To address this gap, in this study, we examined the patterns of informant discrepancies in the Chinese cultural context, aiming to provide a more comprehensive and nuanced understanding of informant discrepancies in Chinese families.

### 1.2 Informant discrepancies and problem behaviors

#### 1.2.1 Associations between quantitative features of informant discrepancies and problem behaviors

The discrepancy–maladaptive hypothesis posits that parent–adolescent discrepancies in parenting, reflecting problems in family functioning, may be maladaptive for adolescent development [17]. These discrepancies indicate the lack of communication between parents and adolescents and high levels of conflict [28,41]. Under such circumstances, parents are more likely to overlook their adolescent's daily life, whereabouts, and activities, which hampers their ability to protect the adolescent from harm or reduce the possibility of their teenager child engaging in problematic behaviors [17].

Further support for the aforementioned hypothesis linking informant discrepancies to problem behaviors in adolescents can be found in prior studies. Parent–adolescent discrepancies in positive parenting have been associated with increased reports of HIV risk behaviors, risky sexual behaviors, and drug and alcohol use among adolescents in Hispanic–American families [29,42]. In Dutch families, discrepancies in warmth and discipline were associated with higher aggressive and rule-breaking behaviors in adolescents [31]. Similar outcomes were discovered in two large-scale longitudinal studies, where discrepancies in monitoring resulted in more adolescents reporting delinquent behaviors [34].

Most existing studies have been conducted in Western countries, with those in mainland China nearly lacking. Studies in the Chinese context are crucial because of the country's large population and its cultural traditions, which are distinct from those of Western countries [43,44]. As mentioned before, parent–adolescent disagreements may be more pronounced in the Chinese context, potentially contributing to more severe problem behaviors [22]. Specifically, the less verbal expression of love or open-discussion affection between parents and adolescents resulted in more ineffective communication and, finally, may enlarge parent–adolescent disagreements [23,28]. In addition, existing studies stressed that informant discrepancies were responsible for adolescents' maladaptive behaviors [18,27,29]. For example, adolescents perceiving lower level of parental monitoring and warmth resulted in severe depression, anxiety, and delinquent behaviors [21,27,34]. Hence, to better understand the association between informant discrepancies and problem behaviors among adolescents, particularly among secondary vocational school students in families based in mainland China, this topic needs further investigation.

Existing studies have overemphasized the direct relationship between informant discrepancies and problem behaviors, while investigations into the underlying mechanisms are scarce, and fewer studies have explored potential mediators. Yeung and colleagues [45] observed the relationship between informant discrepancies and child perspective mediated by self-concept; however, they did not consider informant discrepancies and problem behaviors. Therefore, it is crucial to further investigate the mechanisms underlying the relationship between informant discrepancies and problem behaviors.

Self-control is “the ability to override or change one's inner responses, as well as to interrupt undesired behavioral tendencies and refrain from acting on them” [46]. Existing studies have demonstrated the associations between self-control and informant discrepancies or problem behaviors. Specifically, informant discrepancies are negatively associated with individuals' self-control [45,47]; the greater the discrepancies, the lower is the level of self-control. Furthermore, numerous studies have established a link between self-control and problem behaviors, indicating that low self-control is associated with more severe problem behaviors [48,49]. However, no empirical studies have revealed the mediation role of self-control in the relationship between parent-adolescent discrepancies in positive parenting styles and adolescent problem behaviors.

Some theories could support the mediating effect of self-control in the association between informant discrepancies and problem behaviors. Cognitive dissonance theory stressed when adolescents' perceptions of parenting differ from those of their parents, cognitive conflicts such as discomforting feelings or conflicting attitudes may arise. To reduce dissonance, youngsters must actively adjust their beliefs, values, psychological states, and behaviors for cognitive consistency [50]. However, this maladaptive alteration process may impede growth, particularly undermining self-control and ultimately exacerbating internalizing and externalizing problems [47]. Furthermore, the general strain theory (GST) and self-control theory (SCT) highlight that parent–adolescent discord, like informant discrepancies, undermines youngsters' self-control, thus leading to problem behaviors [51]. Another possible explanation is that parent-adolescent discrepancies in parenting styles may lead to more conflict, which reflects family malfunction [17]. Under this circumstance, adolescents paying attention to dealing with the conflict may exhaust their cognitive resources according to the limited-resources theory, which would not be beneficial to developing good self-control ability and thus lead to problem behaviors [52]. Hence, we propose that adolescents' self-control may mediate the relationship between informant discrepancies and internalizing and externalizing problems.

#### 1.2.2 Associations between patterns of informant discrepancies and problem behaviors

Few studies have specifically examined different patterns of informant discrepancies, and even fewer have explored the association between specific patterns of informant discrepancies and problem behaviors. Based on the discrepancy scores in parental sacrifice, Leung [38] categorized participants into four patterns by using hierarchical cluster analysis. No studies have, however, compared problem behavior scores across subgroups. De Los Reyes and colleagues [20] and Hou and colleagues [27] focused on contrasting the levels of adolescents' negative outcomes (e.g., delinquent behaviors, depressive symptoms, and anxiety) across different profiles. LPA was used to divide participants into three patterns, and the study results revealed that the parent consistently over child profile exhibited significantly higher levels of depression, anxiety, and delinquent behaviors among adolescents. This suggests that when parents perceive higher levels of behavioral control than their offspring, they may lack awareness of their children's whereabouts and activities, which impedes their ability to protect their adolescent children from harm or reduces the likelihood of their children engaging in problematic behaviors.

Contemporary studies have primarily focused on the quantitative aspects of informant discrepancies and their association with problem behaviors, providing limited information regarding other discrepancy patterns and their effects on problem behaviors [32]. Therefore, exploring the connections between adolescent–parent discrepancies and adolescent maladjustment, with specific emphasis on different patterns of informant discrepancies [32], is imperative to obtain valuable insights into the interventions for addressing adolescent problem behaviors. The present study aims to contribute to these critical areas.

### 1.3 The current study

Based on the above review, this multi-informant study aims to better understand the relationships between parent–adolescent discrepancies in positive parenting (care and behavioral control) and internalizing and externalizing problems. The study is based on the discrepancy–maladaptive hypothesis [17], GST [51], and SCT [51].

First, we explored the characteristics of parent–adolescent discrepancies in positive parenting. Building upon previous literature [21,22,39], we present Hypothesis 1, which posits that among secondary vocational school students, adolescents' perceived care and behavioral control is significantly lower than their parents.

Second, from the quantitative perspective, we examined the associations between informant discrepancies and problem behaviors and the mediating role of self-control in this relation. The parent-adolescent discrepancies in parenting style and adolescents' development may differ from existing Western studies because of the variant cultural backgrounds. According to the cognitive dissonance theory, GST, and CST, self-control may mediate the relationship between informant discrepancies and adolescents' problem behaviors. In addition, secondary vocational school students are a minority group who are labeled as “underachievers” in mainstream education and encounter more stress, resulting in more sensitivity to problem behaviors [53,54]. Given the social context in China, therefore, it is crucial to investigate the relationship between informant discrepancies in parenting styles and problem behaviors among secondary vocational school students. Drawing on the existing literature and theoretical frameworks [21,29,39,42], we present Hypothesis 2, which posits that informant discrepancies are positively associated with higher levels of internalizing and externalizing problems among these students. Hypothesis 3, based on GST and SCT, proposes that self-control mediates the relationship between informant discrepancies and adolescent problem behaviors.

Third, we investigated the discrepancy profiles and their association with problem behaviors, focusing on the qualitative perspective. In particular, adolescents perceiving positive parenting lower than parents have been reported to exhibit significantly higher problem behavior scores [20,27]. Building on this literature [20,27], we hypothesized the existence of three distinct profiles, namely nearly no difference, adolescents perceive lower than parents, and adolescents perceive higher than parents profiles; only the adolescents perceive lower than parents profile resulted in more internalizing and externalizing problems (Hypothesis 4).

## 2 Method

### 2.1 Participants and procedures

We recruited 502 adolescent–parent dyads from five secondary vocational schools in Jiangsu Province, China. The inclusion criteria were as follows: (1) aged between 15 and 18 years, (2) belonging to intact families, (3) provided consent to participate in the study (including parental approval), and (4) carefully completed the questionnaires, that is, did not give the same response for every question, had relative speed index >2, and maintained a non-response rate of <10 % [22,38,55]. After eliminating the participants who did not meet the specified criteria and the unmatched questionnaires, 349 pairs of questionnaires were retained for analysis. The main caregivers for the adolescents were 162 fathers and 187 mothers. Most of the parents (60.2 %) graduated from middle schools and were employed in agriculture or industrial production. The demographic information of adolescents is presented in Table 1. The standardized scores of families' socioeconomic status (SES) were calculated using the following procedure. According to the PISA 2009 guidelines [56], we employed three indicators—income, parents' occupation, and parents' education level—to assess SES. Due to the confidential nature of income in Chinese families, direct surveys on income tend to be prone to inaccuracies. Therefore, we utilized the family affluence scale (FAS) to measure family income. The FAS consists of five items, each requiring a “Yes” or “No” response. The FAS scale can comprehensively represent income among Chinese families [57]. For parents' occupation, we used the 10 occupational classes established by Lu [58], which aligned with the Chinese occupational landscape. Each job was assigned a score from 1 to 10. For parents’ education level, we considered six classes with scores from 1 to 6. The adolescents were required to complete all above scales.

**Table 1:** Demography information for adolescents.

	Grade 10	Grade 11
Boys (age)	94 (*M* = 15.81 years, *SD* = 0.39 years)	69 (*M* = 16.89 years, *SD* = 0.42 years)
Girls (age)	111 (*M* = 15.91 years, *SD* = 0.42 years)	75 (*M* = 16.78 years, *SD* = 0.45 years)

After invalid questionnaires were excluded, we computed the SES indices through a three-step process. First, we compared the occupation scores of the parents in each family and selected the higher score^3^ as the occupation index. Similarly, we compared the education scores of the parents in each family and considered the higher score as the education index. The total FAS score was used as the family income index. Second, we excluded families with missing responses in at least two of the three socioeconomic indices and then employed the expectation-maximization (EM) algorithm to impute the missing data. Subsequently, we transformed these socioeconomic indices into *z*-scores. Third, we conducted principal component analysis on the three standardized socioeconomic indices to determine the factor loadings. The SES index was calculated using Eq. (1):

(1) SES = (β_1_ * Z_Education_ + β_2_ * Z_Occupation_ + β_3_ * Z_Income_) / ε_f_

Where β_1_–β_3_ represent factor loadings of the occupation, education index, and family income index, respectively, and ε_f_ represents the eigenvalue of the first factor in the principal component analysis. Hence, the standardized family SES scores in this study were between −2.71 and 3.91.

After obtaining necessary approvals, we visited the schools and administered questionnaires. Before testing, we re-emphasized that the data would remain anonymous for research purposes and that the participation was voluntary. No adolescents or parents we approached declined to participate. Adolescents completed the paper-pencil questionnaires during regular class time under our guidance. First, they rated the parenting styles, and after two weeks, they completed the self-control and brief problem monitor scales. Simultaneously, the parents were requested to complete an online survey regarding parenting styles. All procedures involving human participants adhered to the ethical guidelines outlined by the American Psychological Association and were approved by the Ethic Review Board of Biomedical Studies at Nanjing Normal University (No. NNU202111031).

### 2.2 Measurements

#### 2.2.1 Parental caring and behavioral control

Based on the theory of Maccoby and Martin proposed in 1983 and other existing questionnaires [43,59], items were developed through semi-structured interviews conducted with parents, secondary vocational school students, and teachers. After initial and formal testing, both parent and adolescent versions of the final questionnaire were developed. The self-designed questionnaire encompassed two dimensions, namely caring and behavioral control. The caring dimension comprised 10 items (e.g., “Father/Mother actively respond to my feelings or needs [adolescent version]”/“I actively respond to my child's feelings or needs [parent version]”), and the behavioral control dimension comprised eight items (e.g., “Father/Mother clearly knows my situation in school [adolescent version]”/“I clearly know my child's situation in school [parent version]”). All items were rated on a 7-point Likert scale, ranging from 1 (completely disagree) to 7 (completely agree). In formal testing, the confirmatory factor analysis (CFA) model fitted the data well, χ^2^ ≤ 305.118 (*df* = 134), CFI ≥0.921, RMSEA ≤0.060 (90 % CI ranged from 0.037 to 0.068). The model-based composite reliability ranged between 0.86 and 0.93, and it was significantly correlated with the parental bonding instrument (Chinese version) [60] and adult attachment scale (Chinese version) [61]. We obtained two reports of caring and behavioral control, namely a parent self-report and an adolescent self-report of parents (the main caregiver who participated in the study). All values of Cronbach's α were between 0.85 and 0.93.

#### 2.2.2 Internalizing and externalizing behaviors

Adolescents completed two subscales from the Chinese adaptive version of brief problem monitor [62], internalizing (six items; e.g., “I feel worthless or inferior”), and externalization (seven items; e.g., “I have a hot temper”). Items were rated on a 3-point Likert scale, ranging from 0 (not true) to 2 (often true). The scale is suitable for children and adolescents aged 6–18 years and has good reliability and validity [62]. In this study, Cronbach's α was 0.86 and 0.66 for the internalizing and externalizing behavior scales, respectively. A CFA model with two factors was developed to test the factorial validity of the scale. Using the robust weighted least squares estimator (WLSMV), a fitted well model^4^ was available, χ^2^ = 96.682 (*df* = 63), CFI = 0.989, RMSEA = 0.039 (90 % CI = [0.022, 0.054]).

#### 2.2.3 Self-control

Adolescents completed the Chinese adaptive version of the self-control scale (SCS) [46,63]. The scale comprised 19 items (example of a sample item was “I am self-indulgent at times”). Each item was rated on a 5-point Likert scale, ranging from 1 (not at all) to 5 (very much). The reliability and validity of the adaptive scales were established by Tan and Guo [63]. Cronbach's α was 0.86; a CFA model fitted the data well (χ^2^ = 207.168, *df* = 114, CFI = 0.926, RMSEA = 0.048, RMSEA 90 % CI = [0.038, 0.059]) after the deletion of items 14 and 15 (because factor loadings were <0.40).

### 2.3 Data analysis

Descriptive statistics, correlation analysis, and MANOVA were performed using SPSS 25.0. Other analyses were performed using Mplus 8.0 [64].

First, measurement invariance (MI) of parenting style across informants—which is a prerequisite of latent difference score (LDS) model—was evaluated using multi-group CFA. MI was used to compare increasingly restrictive models, reflecting configural (M0), metric (M1), and scalar (M2) invariances [34,65]. Because the data were not normally distributed (all *p*s < .001 based on the Shapiro-Wilk test), the MLR was used. In nested model comparisons, a significant increase in Satorra-Bentler χ^2^ or ΔCFI ≥0.01 and ΔTLI ≥0.01 indicated that the more stringent constraint was not supportable [66].

Second, to test Hypothesis 1, informant discrepancies were examined using the LDS model. As proposed by de Haan and colleagues [31], the model used second-order latent variables to assess differences between informants’ perceptions of the same behavior (e.g., self-rating vs. other-rating) [31]. The first-order latent factors were derived from observed variables representing individual informant reports. Subsequently, the second-order latent factor, namely LDS (Δ), was derived from the first-order latent variables representing individual informant reports using Eq. (2):

(2) Y_other-rating_ = 1 * Y_self-rating_ + 1 * Δ_self, other_

The consequences of subtraction were simulated by setting the factor loadings of Y_other-rating_ and Δ_self,other_ to 1. The different score indicated the portion of the Y_other-rating_ score that deviated from the Y_self-rating_ [31,67]. Consequently, the different scores provided information about the divergent views within a dyad (in this study, parent–adolescent) while accounting for the effect of self-rating. The discrepancy scores encompassed means (μ_Δ_), variances ($\sigma_{\Delta }^{2}$), and covariance with the self-rating (σ_Δ-self_). The means of the LDS indicated the direction and extent of informant discrepancies, with a positive LDS mean signifying that the other-rating was higher than the self-rating, while a negative average representing that the other-rating was lower than the self-rating [31,67]. Variances in LDS indicated the extent of discrepancies within dyads across the sample, and the covariance of LDS reflected the relationship between self-rating and the LDS [31,67]. In this study, caring and behavioral control were simultaneously included in the model. Parent-reporting was considered self-rating, whereas adolescent-reporting was considered other-rating. Additionally, the means of parent-reporting were set to 0 for model identification.

Third, a regression model was employed to test Hypothesis 2, with the discrepancy score derived from LDS as the independent variable and the internalizing and externalizing problems as the dependent variables. Structural equation modeling (SEM) was used to investigate Hypothesis 3. A model is considered to fit the data well when fit indices meet the criteria (e.g., CFI ≥0.900, TLI ≥0.900, RMSEA ≤0.080, SRMR ≤0.080).

Fourth, the LPA and MANOVA were conducted to examine Hypothesis 4. LPA was performed on the latent discrepancy scores of parenting styles to identify distinct patterns of homogeneous discrepancies. Latent discrepancy scores were obtained by saving factor scores from the LDS model created in the second step. Subsequently, a set of LPA models, spanning from 1-class to 5-class models, were evaluated using MLR estimation [20,68]. To determine the optimal model, the Akaike Information Criterion (AIC), the Bayesian Information Criterion (BIC), and the adjusted Bayesian Information Criterion (aBIC) were compared between the k-class model and the (k-1)-class model. The lower values of these criteria, the better the model fit [68]. We also considered the Entropy value, with a value > 0.8 representing a classification accuracy >90 % [68]. In addition, the Lo-Mendell-Rubin test (LMR) and the Bootstrap Likelihood Ratio Test (BLRT) were conducted. A significant p-value of the LMR and BLRT between the k-class model and the (k-1)-class model indicated that the k-class model was better than the (k-1)-class model [68]. Despite these statistical indices, focusing on the substantive meaning of the obtained profiles is crucial. Using the profile derived from LPA as the independent variable and internalizing and externalizing problem behaviors as the dependent variables, a MANOVA model was established.

Studies have highlighted differences related to adolescents’ gender and age [13,17,47] and SES [21,39] to the study variables. Hence, these demographic variables were included as covariates.

## 3 Results

### 3.1 Descriptive statistics

Means, standard deviations, Pearson correlation coefficients, skewness, and kurtosis of the study variables are presented in Table 2. Correlation analysis results indicated a weak relationship between adolescent-reported and parent-reported caring and behavioral control. Further, parent- and adolescent-reported caring and behavioral control exhibited positive associations with self-control, and adolescent-reported caring and behavioral control were related negatively internalizing and externalizing behaviors.

**Table 2:** Descriptive statistics and correlations.

	1	2	3	4	5	6	7
**Parent-rated**							
1 Caring	5.98 ± 1.07						
2 Behavioral control	.68**	5.30 ± 1.15					
**Adolescent-rated**							
3 Caring	.04	−.03	5.45 ± 1.13				
4 Behavioral control	−.02	.04	.36**	4.60 ± 1.23			
5 Self-control	.11*	.13*	.14*	.03	3.36 ± 0.65		
6 Externalizing	−.03	.04	−.16**	.02	−.59**	0.40 ± 0.31	
7 Internalizing	.01	.06	−.23**	−.04	−.46**	.54**	0.60 ± 0.50
Skewness	−2.00	−1.11	−1.13	−0.39	0.11	0.74	0.82
Kurtosis	5.10	1.71	1.45	−0.11	−0.26	0.26	0.24

*Note*. On the diagonal is *M* ± *SD*; *p < .05; **p < .01.

### 3.2 Characteristics of parent–adolescent discrepancies in the perceived parenting styles

The outcomes of the MI analysis for adolescents' and parents' reports are presented in Table 3. Model fit indices of the configural invariance model (M0) were acceptable. Based on the M0 model, metric invariance was supported since both ΔCFI and ΔTLI were <0.01. However, based on the M1 model, scalar invariance could not be established. Therefore, the constraints on parenting style items 4, 5, 9, 10, 11, and 14 across informants were relaxed, resulting in the establishment of a partial scalar invariance model [69].

**Table 3:** Results of measurement invariance.

Model	*S*-Bχ^2^	*df*	CFI	TLI	RMSEA [90 % CI]	SRMR	Comparison	ΔCFI	ΔTLI
M0	998.860	570	.916	.908	.046 [.042, .051]	.047			
M1	1032.843	586	.913	.906	.047 [.042, .051]	.056	M1 vs. M0	−.003	−.002
M2	1237.688	604	.876	.871	.055 [.050, .059]	.089	M2 vs. M1	−.037	−.035
M2*	1069.709	596	.908	.902	.048 [.043, .052]	.060	M2* vs. M1	−.005	−.004

*Note*. M0 = configural invariance; M1 = metric invariance; M2 = scalar invariance; M2* = partial scalar invariance.

**Fig. 1:**
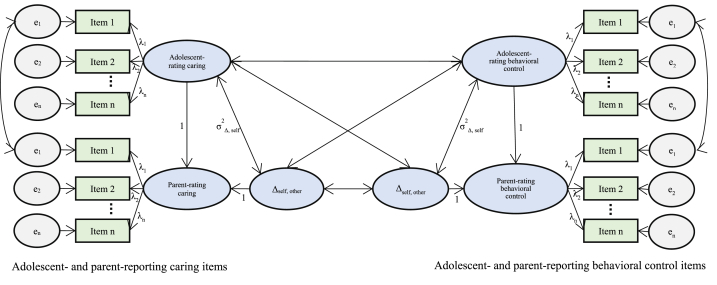
Graphical presentation of a latent difference score model *Note*: Similar labels for the factor loadings and intercepts denote constraints. For clarity, correlations between residual variances of the same items across informants were not provided.

After confirming the MI across informants, we tested Hypothesis 1 by establishing the LDS model, with the parent reports as self-rating and the adolescent reports as other-rating [31]. As mentioned, caring and behavioral control dimensions were simultaneously included in one model and the parent-adolescent discrepancies were estimated within each dimension (Fig. 1). The model fitted the data well, χ^2^ = 1069.709 (*df* = 596), CFI = 0.908, TLI = 0.902, RMSEA = 0.048 (90 % CI = [0.043, 0.052]), SRMR = 0.060. Parameter estimates are presented in Table 4. The 95 % confidence interval of the LDS means revealed that adolescents tended to underestimate their parents' caring and behavioral control compared with those reported by the parents (negative LDS means). Furthermore, the LDS variances in this sample indicated substantial variations across the parent–adolescent dyads. The negative correlations between parent reports and LDS means suggested that the higher the parents rated themselves on caring and behavioral control, the more the adolescents tended to underestimate these behaviors. In addition, the correlation between parent-reported caring and behavioral control was positive and significant (*r* = 0.72, *p* < .001), implying that increased parent-reported caring was related to higher levels of parent-reported behavioral control. Finally, the informant discrepancies in caring were positively and significantly correlated with parent-adolescent discrepancies in behavioral control (*r* = 0.61, *p* < .001).

**Table 4:**
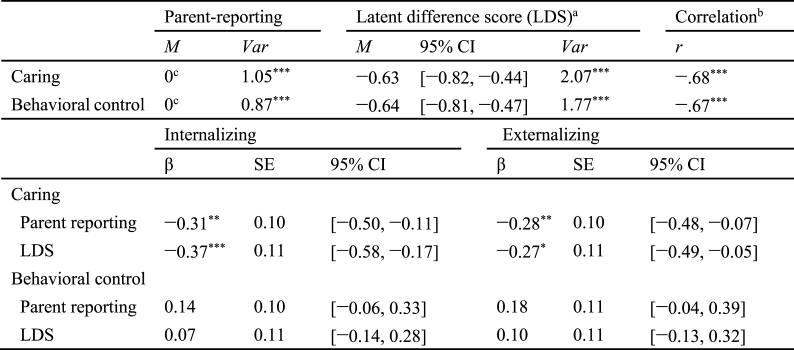
Outcome of latent difference score model and associations between LDS and problem behaviors.

*Note*. ^a^ Second-order latent variable Δ _self, other_; ^b^ correlation between parent-reporting and latent difference score; ^c^ they were set to zero for model identification. *p < .05 **p < .01 ***p < .001.

### 3.3 Discrepancies as predictors of maladjustment and the mediating role of self-control

Using the LDS model, we further explored Hypothesis 2 by incorporating the variables, internalizing and externalizing behavior problems. The model fitted the data well, χ^2^ = 1802.910 (*df* = 1120), CFI = 0.901, TLI = 0.896, RMSEA = 0.042 (90 % CI = [0.038, 0.045]), SRMR = 0.057. Parameter estimates (Table 4) indicated that parent–adolescent discrepancies in caring were positively and significantly associated with internalizing and externalizing problems, suggesting that larger informant discrepancies corresponded to more pronounced problem behaviors. Furthermore, parent-reported caring demonstrated a significant negative relationship with internalizing and externalizing problems, implying that the adolescents whose parents reported higher levels of caring tended to exhibit lower problem behaviors. However, parent self-report behavioral control and parent–adolescent discrepancies in behavioral control were not significantly related to problem behaviors.

**Fig. 2:**
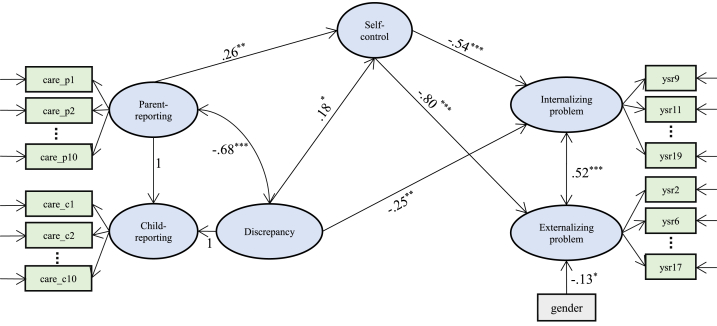
Mediation effect of self-control. *Note*. All coefficients above were standardized; care_px represent items concerning parent-reporting caring; care_cx represent items about adolescent-reporting caring; ysrx represent items regarding adolescent-reporting problem behaviors; discrepancy represents second-order latent variable Δ _self, other_; For clarity, only significant outcomes were shown; *p < .05, **p < .01, ***p < .001.

The relationship between informant discrepancies in behavioral control and problem behaviors was nonsignificant; hence, the mediation role of self-control was only tested in the relationship between parent–adolescent discrepancies in caring dimension^5^ and problem behaviors. Adolescents' gender, age, and family SES were considered as covariates, and a mediation model was constructed to test Hypothesis 3. The model fit the data well, χ^2^ = 1901.288 (*df* = 1302), CFI = 0.905, TLI = 0.900, RMSEA = 0.036 (90 % CI = [0.033, 0.040]), SRMR = 0.055 (Fig. 2). Our results indicated that parent–adolescent discrepancies negatively and significantly predicted self-control, while self-control exhibited a significant negative relationship with problem behaviors. In this model, the direct effect of informant discrepancies on internalizing problems was significant (β = −.25, 95 % CI = [−0.40, −0.09]), suggesting that self-control partially mediated the impact of informant discrepancies on internalizing problems (β_Indirect effect_ = −0.10, 95 % CI = [−0.20, −0.00], accounting for 28.49 % of the total effect). However, no direct effect of informant discrepancies on externalizing problems was observed, implying that self-control mediated the influence of informant discrepancies on externalizing problems (β_Indirect effect_ = −0.15, 95 % CI = [−0.20, −0.00], accounting for 68.92 % of the total effect). Additionally, parent self-reports positively and significantly predicted adolescents' self-control, which subsequently affected adolescents' problem behaviors. This finding suggested that adolescents' self-control mediated the relationship between parent-reported caring and adolescent-reported problem behaviors.

### 3.4 Associations between patterns of informant discrepancies and problem behaviors

**Fig. 3:**
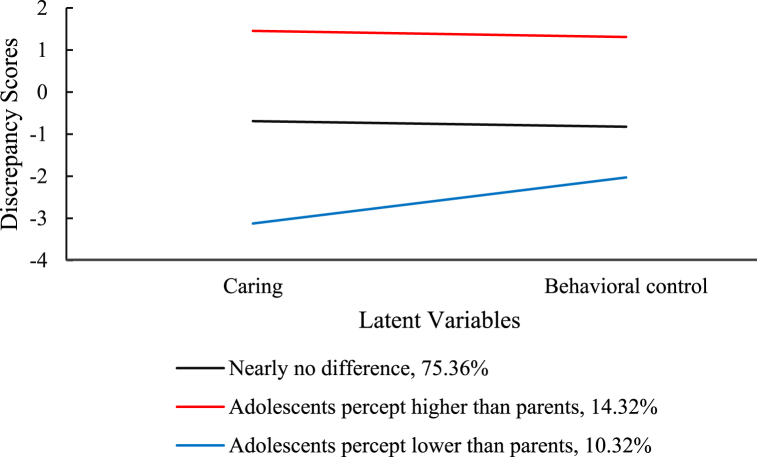
Discrepancy scores for different latent classes.

LPA and MANOVA were conducted to test Hypothesis 4. Following the procedure outlined in the data analysis section, a series of LPA models were established, and the fit indices are presented in Table 5. Considering the interpretability of each profile, although the 5- and 4-class models demonstrated superiority over the rest models, they contained at least one class that lacked coherence. Consequently, the 3-profile solution was selected as the optimal model [68,70]. Class 1 (75.36 % of participants) was labeled “nearly no difference” because scores on all items were close to zero; Class 2 (10.32 % of participants) was designated “adolescents perceive lower than parents” because scores on all items were <0; Class 3 (14.32 % of participants) was characterized by “adolescents perceive higher than parents” as all items’ scores were >0. Fig. 3 displays the graphic description of the 3-class solution.

**Table 5:** Model fit index of latent profile model.

Model	AIC	BIC	aBIC	Entropy	LMR(*p*)	BLRT(*p*)	Class probabilities
1	2377.57	2393.00	2380.31	–	–	–	1.00
2	2273.08	2300.06	2277.85	.737	.010	<.001	.79/.21
3	2222.37	2260.92	2229.19	.796	.269	<.001	.75/.14/.11
4	2152.55	2202.67	2161.43	.845	<.001	<.001	.66/.21/.10/.03
5	2136.75	2198.44	2147.68	.086	.020	<.001	.63/.20/.13/.03/.01

MANOVA was conducted, with latent profiles as the independent variable, adolescents’ gender, age, and family SES as covariates, and internalizing and externalizing as the dependent variables. The main effect of latent profiles on dependent variables was significant, *F* (4, 686) = 5.95, *p* < .001, $\eta_{p}^{2}$ = 0.03. Specifically, the internalization scores were significantly different across profiles, *F* (2, 343) = 11.27, *p* < .001, $\eta_{p}^{2}$ = 0.06. Post-hoc analysis indicated that the internalization score was higher in Class 2 than in other classes (all *p*s < .001). The internalization scores did not differ significantly between Classes 1 and 3 (*p* = .474). In addition, the externalizing problem scores also varied across profiles, *F* (2, 343) = 4.78, *p* = .009, $\eta_{p}^{2}$ = 0.03, with higher externalization scores in Class 2 than in Class 1 (*p* = .012) and Class 3 (*p* = .016) and no difference between Classes 1 and 3 (*p* = 1.000). These findings suggested that adolescents are more likely to experience internalizing and externalizing problems when they hold more negative perceptions of parenting styles.

## 4 Discussion

The primary objective of this study was to investigate the association between informant discrepancies and problem behaviors in a sample of families with secondary vocational school students [21,27,34,71]. Parents and adolescents from 349 intact families completed the parenting style scale, with adolescents also responding to the SCS and brief problem monitor scale. Informant discrepancies feature both quantitative and qualitative perspectives, both of which were covered in our analysis [32]. From the quantitative perspective, we investigated whether self-control mediated the relationship between informant discrepancies and problem behaviors [45]. From the qualitative standpoint, we examined whether the scores of problem behaviors varied across discrepancy patterns [20]. Our findings revealed that self-control mediated the association between informant discrepancies in caring and problem behaviors, and internalization and externalization scores were higher in the “adolescents' perceive lower than parents” profile.

### 4.1 Characteristics of parent–adolescent discrepancies in perceived parenting styles

Consistent with findings reported in previous studies [22,31], our study revealed that secondary vocational school students tend to perceive lower levels of caring from their parents, thereby supporting Hypothesis 1. This observation can be explained by the generational stake hypothesis, which suggests that parents may overestimate the care they provide to their offspring because of their desire to create a nurturing environment [36]. Additionally, the attribution bias context model emphasizes parents' tendency to perceive their positive parenting behaviors as consistent while attributing any negative parenting behaviors to external factors. Adolescents tending to become more independent may become less receptive to their parents’ meticulous care, thus underestimating the positive parenting they receive [37].

Consistent with Hypothesis 1, secondary vocational school students frequently perceive lower levels of behavioral control than their parents, which may be influenced by cultural factors. Chinese and American families interpret specific parenting philosophies differently. American culture emphasizes on child-centered equality and parental discipline, where restrictions are often viewed negatively. In Chinese families, parental discipline and restrictions on adolescents are common and have been associated with positive outcomes such as adolescents' psychological well-being and academic success [43,44,72]. Considering the generational stake hypothesis and attribution bias context model, it is plausible that adolescents in our study perceived lower behavioral control than their parents.

### 4.2 Discrepancies as predictors of maladjustment and the mediating role of self-control

Parent–adolescent discrepancies in caring are associated with high levels of internalizing and externalizing behavior problems, which is in agreement with Hypothesis 2 and earlier research [21,45]. These results can be explained by the discrepancy-maladaptive hypothesis, which suggests that informant discrepancies reflect parents' limited awareness of their adolescents' conditions and actions, which hinders their ability to prevent adolescents from engaging in harmful behavior.

In contrast to Hypothesis 2, no significant relationship was observed between informant discrepancies in behavioral control and internalizing and externalizing problems. This phenomenon may be attributed to the complex nature of behavioral control, which encompasses various aspects such as parental monitoring, discipline, expectations, and knowledge [43]. Discrepancies in these subdimensions may vary inconsistently. For example, discrepancies between parent- and adolescent-reported discipline were reported to be nonsignificant [73], while Ksinan and Vazsonyi [34] found that mothers perceived higher monitoring than their children. We included all four subdimensions of behavioral control that may have contributed to the lack of association between parent–adolescent discrepancies in behavioral control and problem behaviors. Thus, we did not examine the mediating role of self-control in this context.

Furthermore, we verified Hypothesis 3 by examining the mediating role of self-control in the relationship between parent–adolescent discrepancies in caring and internalizing and externalizing problems. Specifically, informant discrepancies significantly predicted lower self-control, which is consistent with the results reported by Yeung [47]. This suggests that when adolescents perceive less caring than their parents, they tend to avoid communication with their parents, which hinders their ability to acquire effective strategies for improving self-control [47]. Moreover, self-control significantly predicted lower internalizing and externalizing problems, which is consistent with the findings of previous studies [48,49]. Thus, adolescents with weaker self-control are more likely to focus on passive or impulsive events, which hinders their ability to develop effective coping strategies, ultimately leading to internalizing and externalizing problems.

Overall, our study contributes to the existing literature on GST and SCT, which highlight the mediating role of self-control in the relationship between informant discrepancies and adolescents' problem behaviors [51]. When secondary vocational school students perceive lower caring than their parents, communication avoidance and potential parent–adolescent conflicts occur. In these circumstances, parents may lose their temper and engage in scolding or other corrective behaviors, setting a negative example for their adolescent child. According to the attribution bias context model and social learning theory, adolescents are more likely to focus on negative interactions in parent–adolescent relationships [21,30]. Consequently, they may struggle to identify effective strategies for cultivating good self-control under challenging circumstances, leading to more problem behaviors [21,45,49]. In contrast, when the parent–adolescent perception of parenting is consistent, a harmonious atmosphere is created, allowing adolescents to identify positive role models in their parents. They can, consequently, cultivate higher self-control, eventually preventing internalizing and externalizing problems [21].

### 4.3 Associations between patterns of informant discrepancies and problem behaviors

We identified three profiles that align with Hypothesis 4 and earlier research [20,27]. However, the proportions were slightly different than those in previous studies. The largest subgroup was the nearly no difference group (75.36 %), followed by the adolescents perceive higher than parents subgroup (14.32 %). The smallest subgroup was the adolescents perceive lower than parents group (10.32 %). In Chinese culture, harmony is favored, and family strife is discouraged [44]. The influence of rules based on the “five cardinal relations” reinforces children's tendency to conform to their parents' perceptions [44], thus explaining the overwhelming majority of families belonging to the nearly no difference subgroup.

Adolescents who reported lower caring and behavioral control than their parents exhibited severer internalizing and externalizing problems, which is consistent with Hypothesis 4 and the findings of earlier research [20,27]. The outcome provides additional empirical support for the discrepancy–maladaptive hypothesis. When parents perceive their parenting practices more positively than teenagers, they are less likely to recognize any shortcomings in their parenting behaviors and falsely believe they care and know more about their offspring. Under these circumstances, parents may remain unaware of the challenges faced by their adolescent child and thus fail to adapt their parenting behaviors to meet their adolescent children's evolving needs, which may contribute to adolescent maladjustment [17,21,27]. In contrast, other discrepancy patterns might reflect parental behaviors that align with adolescents' increasing desire for autonomy and understanding of their parents, potentially facilitating healthy adjustment in teenagers [17,21,27,74]. Hence, the qualitative aspect of informant discrepancies in parenting sheds light on the discrepancy pattern that is more strongly associated with the severity of problem behaviors.

### 4.4 Limitations, conclusions, and practical implications

It is essential to consider the limitations and caveats of this study. First, cross-sectional data are insufficient to establish clear causal associations between informant discrepancies and problem behaviors [67]. Therefore, future longitudinal studies tracking the same dyads throughout adolescence are necessary to provide more precise conclusions regarding the causal relationships and developmental processes involved. Longitudinal designs will also help determine whether there are reciprocal effects between informant discrepancies and problem behaviors. Second, the effect of school types cannot be tested. This is critical for studying adolescents' psychosocial adjustment as secondary vocational school students encounter more stress than adolescents in normal secondary schools in China – they often face social stigmatization (e.g., bad students go to vocational schools!) [54], which may make them more vulnerable to engaging in problem behaviors [53]. Therefore, future studies should include more individuals featuring diverse school types to enable examination of the potential moderating role of school types. Moreover, the deficit sampling strategies are responsible for the limited generalizability of the conclusion. The current sample was obtained from a particular area in China. Therefore, future studies should recruit participants from variant areas across China to verify the generalizability and robustness of the conclusion under a larger sample size. Finally, although fathers and mothers were included in this study, they were not modeled separately because of the substantially smaller sample sizes of fathers or mothers alone. Future studies should aim to cover larger samples encompassing even more mothers and fathers to validate the present study results.

Despite the above limitations, our study contributes significantly to contemporary literature regarding the associations between informant discrepancies and problem behaviors [32]. To the best of our knowledge, this study is the first to consider both the quantitative and qualitative aspects of informant discrepancies. The findings further advance our understanding of parenting discrepancies, providing valuable insights for future basic research on informant discrepancies by combining the variable-centered method and person-centered modeling technique [32]. Furthermore, our study focused specifically on secondary vocational school students who are often overlooked as a “minority” group in China. Moreover, the study findings shed light on informant discrepancies in parenting within this under-researched population, thereby contributing to the existing literature on parent–adolescent discrepancies by providing critical information that fills a gap in the knowledge base.

The present study findings provide empirical evidence for the discrepancy–maladaptive hypothesis that informant discrepancies in parenting styles are negatively associated with adolescents' problem behaviors from both quantitative and qualitative perspectives. Quantitatively, self-control mediates the relationship between informant discrepancies and problem behaviors, indicating that self-control is a protective factor for the development of problem behaviors in adolescents. Qualitatively, only teenagers in the adolescents perceive lower than parents subgroup demonstrated more problem behaviors. These results convey an unmistakable message that informant discrepancies contribute remarkably to shaping adolescents' problem behaviors. Thus, some practical implications might be articulated. First, based on the degree of informant discrepancies, identifying adolescents who are more vulnerable to experiencing problem behaviors is feasible. Hence, mental health education professionals might utilize this information to tailor appropriate activities and support to prevent the escalation of problems. Additionally, psychiatrists can leverage these findings to develop more efficient and beneficial interventions for reducing the likelihood of problem behaviors among adolescents. Specifically, interventions focused on promoting positive perceptions of caring among teenagers may positively impact their development [27].

## Ethical statement

This study was reviewed and approved by the Ethic Review Board of Biomedical Studies at Nanjing Normal University, with the approval number: NNU202110031.

All participants and their legal guardians provided informed consent to participate in this study.

## Data availability statement

Data used in this research will be made available on reasonable request.

## CRediT authorship contribution statement

**Liuqing Tian:** Writing – review & editing, Writing – original draft, Visualization, Project administration, Methodology, Investigation, Formal analysis, Data curation, Conceptualization. **Cong Xin:** Writing – review & editing, Writing – original draft, Supervision, Conceptualization. **Yuanxia Zheng:** Writing – review & editing, Writing – original draft, Supervision, Conceptualization. **Guoxiong Liu:** Supervision.

## Declaration of competing interest

The authors declare that they have no known competing financial interests or personal relationships that could have appeared to influence the work reported in this paper.

## Footnotes

1 Hereafter, “problem behaviors” referred to “internalizing and externalizing problems,” unless otherwise specified.

2 Namely parent–adolescent discrepancies, hereafter referred to as informant discrepancies or parent–adolescent discrepancies.

3 For example, within a family, if the father's occupation score is five and the mother's is four, we should use the father's score as the occupation index for that family and the same for education level.

4 Since Item 7 (disobedient at home) and 8 (disobedient at school) have similar meanings in Chinese, their residuals are allowed to correlate with each other.

5 In this section, informant discrepancies and parent–adolescent discrepancies specifically refer to discrepancies in caring.
