# Supplementary material for: Parent–adolescent discrepancies in positive parenting and adolescent problem behaviors in Chinese families
Source: Heliyon. 2024 Jan 22;10(3):e25016. doi: 10.1016/j.heliyon.2024.e25016 (PMC10844105; doi:10.1016/j.heliyon.2024.e25016)
Supplement: Multimedia component 1 [file mmc1.docx]

**Appendix I. Questionnaires**

**About the questions**

**(Student Version)**

This booklet asks questions about your life. You are to read them and come to your own conclusions. We sincerely hope you like answering the questions and find them to be fascinating.

Please make an effort to respond to **every inquiry**. Take your time, thoroughly go through everything, and provide the most truthful and correct response you can to each question. **Answers are not right or wrong**. This is **not** an exam. All we want to do is ask how you feel about things.

Please ask the testers if you require assistance understanding any of the questions. Please send it to the tester once you have finished.

**No one**, not even your parent(s), needs to see or **know what you answered**. No one who sees this booklet will be able to identify whose answers they are because **your name and address are not included**. You are free to skip any questions you are not comfortable answering.

Tell the interviewer whether you need assistance reading and answering the questions if you find it difficult to do so on your own.

**Survey 1: Basic Information**

1. Your gender □ Male □ Female
2. When is your birthday (Day) (Month) (Year)
3. Does your family own a car, van or truck? □Yes □No
4. Do you have your own bedroom for yourself? □Yes □No
5. How many computers does your family own? □Yes □No
6. Do you have your own study room for yourself? □Yes □No
7. Does your home have a bathroom? □Yes □No
8. What is your father’s education level?

□Primary education or lower

□Junior secondary

□Senior secondary or secondary vocational education

□Vocational/web-based undergraduate

□Full-time undergraduate

□Postgraduate or higher

1. What is your mother’s education level?

□Primary education or lower

□Junior secondary

□Senior secondary or secondary vocational education

□Vocational/web-based undergraduate

□Full-time undergraduate

□Postgraduate or higher

1. What is your father’s occupation?

□Unemployed or semi-unemployed people

□Agricultural laborers

□Industry workers

□Business services employees

□Individual business owners

□Civil service staff

□Specialist technical staff (e.g., doctor, teacher)

□Private entrepreneurs

□Senior company manager

□State and social managers

1. What is your mother's occupation?

□Unemployed and semi-unemployed people

□Agricultural laborers

□Industry workers

□Business services employees

□Individual business owners

□Civil service staff

□Specialist technical staff (e.g., doctor, teacher)

□Private entrepreneurs

□Senior company manager

□State and social managers

**Survey 2: Your Parent**

The following sentences describe attitudes or behaviors that parents may exhibit. Please choose the number that best matches your perception of your father’s and mother’s behaviors respectively. (Select 7 when totally agree, 1 when totally disagree).

|  |  | **Totally disagree** | **Strongly disagree** | **Slightly disagree** | **Neutral** | **Slightly agree** | **Strongly agree** | **Totally agree** |
| --- | --- | --- | --- | --- | --- | --- | --- | --- |
| (1) My parents will enlighten me when I feel blue | Mother | ① | ② | ③ | ④ | ⑤ | ⑥ | ⑦ |
|  | Father | ① | ② | ③ | ④ | ⑤ | ⑥ | ⑦ |
| (2) When I fail the exam/competition, parents will encourage me to try harder | Mother | ① | ② | ③ | ④ | ⑤ | ⑥ | ⑦ |
|  | Father | ① | ② | ③ | ④ | ⑤ | ⑥ | ⑦ |
| (3) My parents respond actively to my needs or feelings | Mother | ① | ② | ③ | ④ | ⑤ | ⑥ | ⑦ |
|  | Father | ① | ② | ③ | ④ | ⑤ | ⑥ | ⑦ |
| (4) I can count on parents to help me out, if I have some kind of problem. | Mother | ① | ② | ③ | ④ | ⑤ | ⑥ | ⑦ |
|  | Father | ① | ② | ③ | ④ | ⑤ | ⑥ | ⑦ |
| (5) I feel warm when talking to my parents | Mother | ① | ② | ③ | ④ | ⑤ | ⑥ | ⑦ |
|  | Father | ① | ② | ③ | ④ | ⑤ | ⑥ | ⑦ |
| (6) When parents want me to do something, they explain why | Mother | ① | ② | ③ | ④ | ⑤ | ⑥ | ⑦ |
|  | Father | ① | ② | ③ | ④ | ⑤ | ⑥ | ⑦ |
| (7) My parents were patient and attentive when answering my questions | Mother | ① | ② | ③ | ④ | ⑤ | ⑥ | ⑦ |
|  | Father | ① | ② | ③ | ④ | ⑤ | ⑥ | ⑦ |
| (8) When I am ill, my parents will take good care of me | Mother | ① | ② | ③ | ④ | ⑤ | ⑥ | ⑦ |
|  | Father | ① | ② | ③ | ④ | ⑤ | ⑥ | ⑦ |
| (9) I feel relaxed when I talk to my parents | Mother | ① | ② | ③ | ④ | ⑤ | ⑥ | ⑦ |
|  | Father | ① | ② | ③ | ④ | ⑤ | ⑥ | ⑦ |
| (10) My parents always give me a lot of support when I encounter difficulties | Mother | ① | ② | ③ | ④ | ⑤ | ⑥ | ⑦ |
|  | Father | ① | ② | ③ | ④ | ⑤ | ⑥ | ⑦ |
| (11) My parents clearly know my situation in the school | Mother | ① | ② | ③ | ④ | ⑤ | ⑥ | ⑦ |
|  | Father | ① | ② | ③ | ④ | ⑤ | ⑥ | ⑦ |
| (12) My parents take initiative to understand who my friends are | Mother | ① | ② | ③ | ④ | ⑤ | ⑥ | ⑦ |
|  | Father | ① | ② | ③ | ④ | ⑤ | ⑥ | ⑦ |
| (13) My parents know my activities when I am with my friends | Mother | ① | ② | ③ | ④ | ⑤ | ⑥ | ⑦ |
|  | Father | ① | ② | ③ | ④ | ⑤ | ⑥ | ⑦ |
| (14) My parents have clear rules about how I use my leisure time | Mother | ① | ② | ③ | ④ | ⑤ | ⑥ | ⑦ |
|  | Father | ① | ② | ③ | ④ | ⑤ | ⑥ | ⑦ |
| (15) My parents actively understand how I use leisure time | Mother | ① | ② | ③ | ④ | ⑤ | ⑥ | ⑦ |
|  | Father | ① | ② | ③ | ④ | ⑤ | ⑥ | ⑦ |
| (16) My parents usually know when I have tests and examination | Mother | ① | ② | ③ | ④ | ⑤ | ⑥ | ⑦ |
|  | Father | ① | ② | ③ | ④ | ⑤ | ⑥ | ⑦ |
| (17) My parents have clear expectations about what I can do after school | Mother | ① | ② | ③ | ④ | ⑤ | ⑥ | ⑦ |
|  | Father | ① | ② | ③ | ④ | ⑤ | ⑥ | ⑦ |
| (18) My parents actively understand what I do after school | Mother | ① | ② | ③ | ④ | ⑤ | ⑥ | ⑦ |
|  | Father | ① | ② | ③ | ④ | ⑤ | ⑥ | ⑦ |

Survey 3: Your Distress

Please complete the question according to your performance in the last 6 months. If the behaviors are very obvious, please choose 2, and if the behaviors don’t occur, please choose 0.

|  | **Not accuracy** | **Sometimes have** | **Very obvious** |
| --- | --- | --- | --- |
| (1) Argue a lot | 0 | 1 | 2 |
| (2) Destroy things belonging to others | 0 | 1 | 2 |
| (3) Disobey my parents | 0 | 1 | 2 |
| (4) Disobey at school | 0 | 1 | 2 |
| (5) Feel worthless or inferior | 0 | 1 | 2 |
| (6) Too fearful or anxious | 0 | 1 | 2 |
| (7) Feel too guilty | 0 | 1 | 2 |
| (8) Self-conscious or easily embarrassed | 0 | 1 | 2 |
| (9) Stubborn | 0 | 1 | 2 |
| (0) Have a hot temper | 0 | 1 | 2 |
| (11) Threaten to hurt people | 0 | 1 | 2 |
| (12) Unhappy, sad, or depressed | 0 | 1 | 2 |
| (13) Worry a lot | 0 | 1 | 2 |

Survey 4: Your Custom

Please indicate how much each of the following statements reflects how you typically are.

|  | **Totally disaccord** | **Disaccord** | **Neutral** | **Accord** | **Totally accord** |
| --- | --- | --- | --- | --- | --- |
| (1) I am good at resisting temptation. | ① | ② | ③ | ④ | ⑤ |
| (2) I have a hard time breaking bad habits. | ① | ② | ③ | ④ | ⑤ |
| (3) I am lazy. | ① | ② | ③ | ④ | ⑤ |
| (4) I do certain things that are bad for me, if they are fun | ① | ② | ③ | ④ | ⑤ |
| (5) People can count on me to keep on schedule. | ① | ② | ③ | ④ | ⑤ |
| (6) Getting up in the morning is hard for me | ① | ② | ③ | ④ | ⑤ |
| (7) People would describe me as impulsive. | ① | ② | ③ | ④ | ⑤ |
| (8) I spend too much money. | ① | ② | ③ | ④ | ⑤ |
| (9) I get carried away by my feelings. | ① | ② | ③ | ④ | ⑤ |
| (10) I do many things on the spur of the moment | ① | ② | ③ | ④ | ⑤ |
| (11) People would say that I have iron self-discipline | ① | ② | ③ | ④ | ⑤ |
| (12) Pleasure and fun sometimes keep me from getting work done | ① | ② | ③ | ④ | ⑤ |
| (13) I have trouble concentrating. | ① | ② | ③ | ④ | ⑤ |
| (14) I am able to work effectively toward long-term goals | ① | ② | ③ | ④ | ⑤ |
| (15) Sometimes I can’t stop myself from doing something, even if I know it is wrong. | ① | ② | ③ | ④ | ⑤ |
| (16) I often act without thinking through all the alternatives | ① | ② | ③ | ④ | ⑤ |
| (17) I lose my temper too easily. | ① | ② | ③ | ④ | ⑤ |
| (18) I often interrupt people | ① | ② | ③ | ④ | ⑤ |
| (19) I sometimes drink or use drugs to excess. | ① | ② | ③ | ④ | ⑤ |

**(Parent Version)**

This questionnaire asks about your strategies to nurture your offsprings. You are to read them and come to your own conclusions. We sincerely hope you like answering the questions and find them to be fascinating.

Please make an effort to respond to **every inquiry**. Take your time, thoroughly go through everything, and provide the most truthful and correct response you can to each question. **Answers are not right or wrong**.

Please ask the testers if you require assistance understanding any of the questions. Please send it to the tester once you have finished.

**No one** needs to see or **know what you answered**. No one who sees this booklet will be able to identify whose answers they are because **your name and address are not included**. You are free to skip any questions you are not comfortable answering.

Tell the interviewer whether you need assistance reading and answering the questions if you find it difficult to do so on your own.

**Survey: Your ways to nurture your child**

The following sentences describe attitudes or behaviors that you may exhibit. Please choose the number that best matches the behavior of yours. (Select 7 if you totally agree, 1 if you totally disagree)

|  | **Totally disagree** | **Strongly disagree** | **Slightly disagree** | **Neutral** | **Slightly agree** | **Strongly agree** | **Totally agree** |
| --- | --- | --- | --- | --- | --- | --- | --- |
| (1) I will enlighten my child when he/she feels blue | ① | ② | ③ | ④ | ⑤ | ⑥ | ⑦ |
| (2) When my child fails the exam/competition, I will encourage he/she to try harder | ① | ② | ③ | ④ | ⑤ | ⑥ | ⑦ |
| (3) I can respond actively to my child's needs or feelings | ① | ② | ③ | ④ | ⑤ | ⑥ | ⑦ |
| (4) My child can count on me to help him/her out, if my child has some kind of problem. | ① | ② | ③ | ④ | ⑤ | ⑥ | ⑦ |
| (5) I feel warm when talking to my child | ① | ② | ③ | ④ | ⑤ | ⑥ | ⑦ |
| (6) When I want my child to do something, I will explain why | ① | ② | ③ | ④ | ⑤ | ⑥ | ⑦ |
| (7) I was patient and attentive when answering my child's questions | ① | ② | ③ | ④ | ⑤ | ⑥ | ⑦ |
| (8) When my child is ill, I will take good care of him/her | ① | ② | ③ | ④ | ⑤ | ⑥ | ⑦ |
| (9) I feel relaxed when I talk to my child | ① | ② | ③ | ④ | ⑤ | ⑥ | ⑦ |
| (10) I always give my child a lot of support when he/she encounters difficulties | ① | ② | ③ | ④ | ⑤ | ⑥ | ⑦ |
| (11) I clearly know my child's situation in the school | ① | ② | ③ | ④ | ⑤ | ⑥ | ⑦ |
| (12) I take initiative to understand who are my child’s friends | ① | ② | ③ | ④ | ⑤ | ⑥ | ⑦ |
| (13) I know my child's activities when he/she is with him/her friends | ① | ② | ③ | ④ | ⑤ | ⑥ | ⑦ |
| (14) I have clear rules about how my child can use his/her leisure time | ① | ② | ③ | ④ | ⑤ | ⑥ | ⑦ |
| (15) I actively understand how my child can use his/her leisure time | ① | ② | ③ | ④ | ⑤ | ⑥ | ⑦ |
| (16) I usually know when my child has tests and examination | ① | ② | ③ | ④ | ⑤ | ⑥ | ⑦ |
| (17) I have clear expectations about what my child can do after school | ① | ② | ③ | ④ | ⑤ | ⑥ | ⑦ |
| (18) I actively understand what my child does after school | ① | ② | ③ | ④ | ⑤ | ⑥ | ⑦ |

The allocated ID was (Please enter the ID we told you before the testing)

**Appendix II. The consent forms**

Dear parents,

Welcome to participate in the research held by the Child Emotional Development and Socialization Lab in the School of Psychology of Nanjing Normal University! This project seeks to reveal the general pattern of adolescents’ growth process, which helps adolescents grow up healthily and improve parent-adolescent interaction.

1. **Introduction**

Parenting style refers to the methods and strategies used by parents in nurturing their children, which are relatively stable, and parents and adolescent children may hold divergent opinions on parenting style. Self-control refers to individuals’ ability to discipline and manage their behaviors and emotions according to the social norm or their own wishes. Problem behaviors mainly include internalizing and externalizing problems. The former mainly refers to anxiety, depression, withdrawal, etc., and the latter mainly refers to aggression, violation of discipline, etc., which can hinder the healthy growth of adolescents. This project is dedicated to exploring the associations between parent-adolescent discrepancies in parenting style and adolescents’ internalizing and externalizing behaviors. The study will help parents to understand adolescents’ behavior and enlighten people to find effective ways to promote adolescent health.

1. **How to participate in**

During the period from Oct 10, 2021, to Oct 31, 2021, your offspring will receive a booklet, and they should spend about *20 minutes* finishing it according to the guidance of the tester (Including surveys about family background, parenting styles, problem behaviors and self-control). At the same time, you will receive a questionnaire link from the classroom chat group on WeChat. You should spend about *five minutes* fill out the questionnaire and submit it via the Internet.

1. **Payments**

If you and your offspring participated in the research, you and your offspring can get ￥5 and ￥10, respectively.

1. **Your Rights**

You can ***withdraw from the survey at any time without any reason***. However, this will result in losing benefits (the minimum payment is ￥2). If you feel uncomfortable or have any questions, please feel free to contact us at any time. We are pleased to provide help.

1. **Our Commitment and Contact Information**

The use of data from the survey is strictly supervised by the Ethic Review Board of Biomedical Studies at Nanjing Normal University. We ensure that the data will be only used anonymously for research at the group level. Please feel free to contact us if you have any questions via the email address [915989079@qq.com](mailto:915989079@qq.com).

1. **Your decision**

Please select your answer:

□ I voluntarily **agree** for **my offspring and me** to participate in this research! I understand and obey the procedure of the survey. I and my offspring can ask any questions to the researchers. I and my offspring can withdraw the survey at any time without any reason. I voluntarily **agree** the data will be only used anonymously for research.

□ I **only agree for me**, but **not my offspring**, to participate in this research.

□ I **disagree** with participating in this research.

Your signature: Date:

I am the □mother/□ father/□ other caregivers (please note: ) of my offspring.

*Please tell us your offspring’s name so we can rule out them if you do not agree with your child to participate in the research (Choose Box 2)*

Your offspring’s name (only you choose the second box above):

Researcher signature: Date:
